# Effect of Natural Sorbents in the Diet of Fattening Pigs on Meat Quality and Suitability for Processing

**DOI:** 10.3390/ani11102930

**Published:** 2021-10-10

**Authors:** Mateusz Ossowski, Łukasz Wlazło, Bożena Nowakowicz-Dębek, Mariusz Florek

**Affiliations:** 1 Department of Animal Hygiene and Environmental Hazards, University of Life Sciences in Lublin, Akademicka 13, 20-950 Lublin, Poland; mateusz.ossowski@up.lublin.pl (M.O.); bozena.nowakowicz@up.lublin.pl (B.N.-D.); 2 Department of Quality Assessment and Processing of Animal Products, University of Life Sciences in Lublin, Akademicka 13, 20-950 Lublin, Poland; mariusz.florek@up.lublin.pl

**Keywords:** natural sorbent, pig, meat, quality

## Abstract

**Simple Summary:**

Animal production generates emissions of gaseous pollutants to the environment. Natural sorbents are used to reduce emissions increase feed safety and reduce the impact of farms on the environment. Therefore, the aim of the study was to assess the effect of three natural sorbents added to the diet of pigs on the composition and physicochemical properties of two skeletal muscles (*Longissimus* *lumborum* and *semimembranosus*) of crossbred pigs. The experiment was carried out on a farm in two production cycles. The animals received feed supplemented with three different natural sorbents. The results indicate that the sorbents used in the diet of pigs have no negative effect on the physicochemical properties of meat or its use as case-ready meat or material for processing.

**Abstract:**

The effect of three natural sorbents added to the diet of pigs on the composition and physicochemical properties of two skeletal muscles—the *musculus* *Longissimus* *lumborum* (MLL) and *musculus* *semimembranosus* (MSM) of crossbred pigs were evaluated. The experiment was carried out on a farm in two production cycles. The addition of biochar (trial 1) significantly influenced instrumental color parameters, shear force and energy, and the oxidative stability of the skeletal muscles, while the proximate composition, pH, texture, and water-holding capacity (WHC) parameters did not differ significantly between groups in either of the two muscles. Similarly, no statistical differences were noted in the proximate chemical composition, texture parameters, or WHC of the meat in trial 2. The addition of both sorbents was associated with a significantly (*p* ≤ 0.01) lower content of haem pigments in the MLL and MSM, which was accompanied by a significantly (*p* ≤ 0.05) higher lightness (L*). Moreover, the MLL muscle of the pigs had higher oxidative stability, as well as lower drip loss (DL). In turn, the MSM of pigs had a significantly lower pH compared to the control group, however, the ultimate pH (48 h) in all groups was within the acceptable range (5.50–5.80). Summing up, the sorbents used are a safe ingredient in the diet of pigs, however, there is a need to continue and strengthen this line of research, including the relationships linking the future production goals of pig farming and processing potential in the meat industry with current climate policy.

## 1. Introduction

According to data from the United States Department of Agriculture (USDA), the world leaders in pig farming and pork production are China and the European Union (EU), [1]. To ensure high product quality, the meat industry and livestock farmers must implement systems guaranteeing the quality and repeatability of the raw meat used in production [2,3,4]. Pig farmers focus primarily on optimal feeding, which is the most important factor determining the economic profitability of production. The quality and safety of feed play an important role in animal production because they determine the magnitude of losses incurred and the health safety of the food product. This necessitates the search for and implementation of methods to improve animal health and productivity. One means of meeting these requirements is feed supplementation with clay minerals, i.e., natural adsorbents, such as zeolite or montmorillonite, which have the capacity to bind and/or adsorb mycotoxins [3,5,6].

The production of animals for meat generates emissions of gaseous pollutants, including greenhouse gases. Livestock farmers are required to implement clean safe techniques (best available techniques—BAT) that reduce pressure on the environment. It is estimated that about 80% of emissions of ammonia (NH_3_) from European agriculture are from animal waste on farms. There is enormous variation between individual countries in the level of NH_3_ emissions from various sectors of production [7]. The EU climate policy is aimed at reducing the impact of farms on the environment. Many authors suggest the need for measures aimed at maintaining a balance between farms and the state of the natural environment. For this purpose, farmers use natural sorbents added to feed and litter that both increase feed safety and reduce the impact of the farms on the environment. Among sorbents of natural origin, the most common are silicates: sodium bentonite, zeolite, halloysite, perlite, or vermiculite. They have a high sorption capacity and are not harmful to animals. Their presence in feces contributes to the reduction in gaseous emissions [8,9,10,11,12,13,14]. Mineral sorbents (zeolites and bentonites) are used in pig farming mainly as feed additives for animals in various age groups: zeolites in the amount of 0.5% to 8% [15] and from 0.5% to 2% in the case of bentonites [16].

In terms of technological quality, the most important characteristics of pork include its chemical composition (including the proportion of intramuscular fat), pH, color, and water-holding capacity (WHC). Instrumental measurement of physicochemical properties makes it possible to identify the direction of changes taking place in meat after slaughter, to diagnose quality defects—most commonly pale soft exudative (PSE) and acid meat and determine an appropriate type of processing [17]. 

The literature, however, lacks studies on the effect of the addition of natural sorbents to feed on the physicochemical properties of pork. For this reason, the aim of the study was to assess the effect of three natural sorbents added to the diet of crossbred fattening pigs on the composition and physicochemical properties of two skeletal muscles.

## 2. Materials and Methods

### 2.1. Experimental Design

The study was conducted on total of 1200 weaners from multi-breed crosses of the Choice Genetics line. All pigs (female and male) were free of the halothane-sensitivity (n) and RN^−^ alleles. The experiment was carried out on a single farm in two consecutive production cycles (experiment 1 and experiment 2), and animals were divided at random into five groups (two control and three experimental). Each group was comprised of 240 individuals kept in three pens with 80 animals per pen. The experimental groups were designated as A, B, and D, and the control groups as C1 and C2. Animals received standard feed for their age group (grower and then finisher). Experiment 1 was begun in winter. The animals in experimental group D received standard feed with a 0.5% addition of biochar, while the control group (C1) received a standard feed. Experiment 2 was begun in the summer. The animals in the two experimental groups (A and B) received feed with the addition of 1.5% composed sorbents (patent application No. P.432877), while the control group (C2) received a standard feed. Sorbents A and B are prepared in the form of granules using a specific granulation and mixing technology. The appropriate weight proportions of aluminosilicates in the mixtures are essential, prevalence of bentonite in group A and of zeolite in group B.

### 2.2. Animal Diets

The ingredients of the diets are presented in Table 1 and Table 2. The nutritional value and content of vitamins and minerals were in compliance with recommendations of the National Research Council (NRC), [18]. In both experiments, the animals were fed ad libitum with free access to drinking water. The animals were under the care of a veterinarian employed at the farm. The results of measurements of the microclimatic conditions in the production buildings, performed regularly throughout the experiment, at no time exceeded the limits established for ensuring animal welfare [19,20]. All tests on animals were performed with the approval of the Local Ethics Committee for experiments on animals (approval no. 100/2015).

### 2.3. Sampling of Experimental Material

Six pigs (three male and three female) were selected for slaughter from each group. The average body weight (BW) of the pigs was as follows: group C1 107.1 ± 3.5 kg, group D 106.5 ± 6.1 kg, C2 107.9 ± 4.1 kg, group A 108.6 ± 2.7 kg, and group B 105.4 ± 5.1 kg. The pigs from experiment 1 were slaughtered in June and the ones from experiment 2 in December. The pigs were transported to the slaughterhouse and slaughtered in accordance with Council Regulation (EC) No. 1/2005 of 22 December 2004 [21] and Council Regulation (EC) No. 1099/2009 of 24 September 2009 [22], respectively. The research material comprised: *m. Longissimus lumborum* (MLL) and *m. semimembranosus* (MSM), which were collected during carcass fabrication after 24 h chill time. Each muscle was divided into three sections of equal weight (approx. 250 g), which were randomized for each analysis (chemical analysis, physicochemical measurements, and texture analysis). Then, the samples were separately vacuum-packed in polyamide and polyethylene (PA/PE) bags with a high gas barrier and a 98% vacuum level and stored at 4 ± 1 °C until analysis.

### 2.4. Physicochemical Measurements

Muscle pH was determined using an ERH-12-6 (Hydromet, Gliwice, Poland) combination glass electrode and a CP-401 waterproof pH meter (Elmetron, Zabrze, Poland) equipped with a temperature sensor and calibrated using buffers with pH 4 and pH 7 ± 0.02 at 20 °C. The water activity (a_W_) of the muscle tissue was measured using the HygroLab C1 (Rotronic, Bassersdorf, Switzerland). Measurements were made in AWQ mode with stabilization set to 15 min after the samples had reached room temperature. The average pH and a_W_ for the sample were calculated from three replicates. Objective color measurements of the skeletal muscles were made using a CR-310 color meter (Minolta Camera Co. Ltd., Osaka, Japan). After 30 min of blooming at 2 °C, the following parameters were measured on the exposed cross-section of the muscle: L*—lightness; a*—red and b*—yellow [23]. The average from three replications was calculated. The total content of haem pigments was determined according to Hornsey [24] using a Varian Cary 300 Bio spectrophotometer (Varian Australia Pty Ltd., Mulgrave, Australia), at a wavelength of 640 nm, calculating the concentration of haematin in μg/g of meat. The oxidative stability of lipids was determined according to Witte et al. [25], using a Varian Cary 300 Bio spectrophotometer at a wavelength of 530 nm. The thiobarbituric acid reactive substance (TBARS) value was expressed in mg of malondialdehyde (MDA) in 1 kg of meat. 

Texture parameters were measured according to Florek et al. [26] using the Zwick/Roell Proline B0.5 (Zwick GmbH & Co., Ulm, Germany) single-column testing machine on meat samples used to assess cooking loss (CL). A Warner-Bratzler V-blade was used to measure the shear force (W-B SF, N) and shear energy (W-B SE, mJ). The measurements were made on muscle sections 4–5 cm in length with a cross-sectional area of 1 cm^2^. The average for the sample was calculated from six replicates. TestXpert^®^ II software (Zwick GmbH & Company KG, Ulm, Germany) was used to analyze the measurement results. For the texture profile analysis (TPA), samples of 20 × 20 × 20 mm were compressed twice to 50% height with a die 70 mm in diameter. TestXpert^®^ II software was used to calculate the mean from three replicates for hardness, springiness, gumminess, and chewiness. 

Drip loss (DL) was determined based on the difference between the weight of the sample before and after storage at 4 °C for 24 h. CL was determined based on the difference between the weight of the sample pre and post heat treatment in a water bath. Meat samples weighing about 75 ± 5 g, sealed in plastic bags, were heated at 70 °C for 45 min (HENDI sous-vide system GN 1/1, Rhenen, Netherlands) and then cooled under running water for 30 min and stored at 4 °C. The amount of free water (mg) was determined according to Grau and Hamm [27] using Whatman No 1 filter paper and a 300 mg weighted sample, applying a constant force of 2 kg for 5 min. The surface of the meat sample (M) and the total loss (T) were measured (in cm^2^) using MultiScan Base image analysis software ver. 14 and expressed as M/T × 100. The average values of water holding capacity parameters were calculated from two repetitions of a single sample.

The proximate chemical composition of meat samples was determined by reference methods. Moisture content was determined by the oven-dry method at 103 °C (Memmert UF30, Schwabach, Germany) according to PN-ISO 1442:2000 [28]; ash content by incineration at 550 °C (Heraeus M110, Hanau, Germany) according to PN-ISO 936:2000 [29]; total protein (N × 6.25) by the Kjeldahl method using a Büchi B-324 distillation unit (Büchi Labortechnik AG, Flawil, Switzerland) according to PN-75-A-04018:1975/Az3:2002 [30]; and free fat by the Soxhlet method (using n-hexane as a solvent) with a Büchi B-811 (Büchi Labortechnic AG, Flawil, Switzerland) according to PN-ISO 1444:2000 [31]. Feder’s number (the moisture to protein ratio—M/P), characterizing the degree of hydration of muscle proteins, was calculated from the moisture content and the crude protein content. The energy value, expressed in kcal per 100 g of muscle tissue, was calculated using the following energy equivalents: 4 kcal for 1 g protein, 9 kcal for 1 g fat [32].

### 2.5. Statistical Analysis

Statistical analysis of the results, performed using Statistica software version 13 (TIBCO Software Inc., Palo Alto, CA, USA), was based on a one-way analysis of variance to estimate the effect of the feeding group on the physicochemical properties of each skeletal muscle. The significance of differences between means for groups was determined by Student’s *t*-test (comparison of two groups in experiment 1) or Tukey’s honest significant difference (HSD) test (comparison of three groups in experiment 2).

## 3. Results

### 3.1. Experiment 1

The proximate chemical composition, pH, texture parameters, and WHC parameters did not differ significantly between groups in either of the two muscles (Table 3). Among physicochemical properties, the effect of the addition of biochar (group D) significantly influenced instrumental color parameters, shear force and energy, and the oxidative stability of the skeletal muscles. The MLL of the group D pigs had significantly (*p* ≤ 0.01) lower content of haem pigments (37.2 vs. 45.8 µg/g), which may have contributed to the significantly (*p* ≤ 0.01) lower L* value (59.34 vs. 55.89) and oxidative stability of the muscle tissue (0.181 vs. 0.228 mg MDA/kg). However, the MLL of this group had significantly higher water activity in comparison to group C1 (0.957 vs. 0.948), and greater hardness expressed as higher shear force (37.9 vs. 27.4 N) and shear energy (117.3 vs. 93.0 mJ) in the W-B test. Similar significant relationships were noted for the MSM of the pigs in group D. In comparison to group C1, it had a significantly lower content of haem pigments (46.2 vs. 62.9 µg/g, *p* ≤ 0.01), less redness (a* = 20.35 vs. 21.19, *p* ≤ 0.05), and higher oxidative stability (0.159 vs. 0.221 mg MDA/kg, *p* ≤ 0.01), but higher shear force (57.5 vs. 37.3 N, *p* ≤ 0.01) and shear energy (220.7 vs. 117.7 mJ, *p* ≤ 0.01).

### 3.2. Experiment 2

The addition of sorbent A or B to feed only slightly modified the physicochemical quality of the two skeletal muscles (Table 4). No statistical differences were noted in the proximate chemical composition, texture parameters, or WHC of the meat. The addition of both sorbents was associated with a significantly (*p* ≤ 0.01) lower content of haem proteins in the MLL of pigs from groups A (41.8 µg/g) and B (46.7 µg/g) than in group C2 (53.7 µg/g). The lower concentration of pigments was accompanied by significantly (*p* ≤ 0.05) higher L* values for the surface of the meat from groups A (54.05) and group B (53.79) compared to group C2 (50.58). Moreover, the MLL of the pigs in both experimental groups had higher oxidative stability (0.238 mg MDA/kg in group A and 0.280 mg MDA/kg in group B), compared to group C2 (0.314 mg MDA/kg), as well as lower DL, amounting to 2.25% (group A) and 2.78% (group B) vs. 3.37% (group C2).

Identical relationships to those observed for the MLL were also noted for the MSM with respect to haem pigments, L*, and TBARS value (Table 2). In addition, the MSM of pigs from both experimental groups had significantly lower pH after 24 and 48 h post-mortem: 5.70 and 5.48 in group A and 5.55 and 5.49 in group B, compared to group C2 (6.02 and 5.79). However, the final pH (48 h) in all groups was within the acceptable range (5.50–5.80).

## 4. Discussion

Mineral sorbents (zeolites and bentonites) are used in pig farming mainly as feed additives for animals in various age groups: zeolites in the amount of 0.5% to 8% [31] and bentonites from 0.5% to 2% [32]. The use of sorbents (including zeolite and montmorillonite) in the diet of fattening pigs has been confirmed to have beneficial effects, improving daily weight gains, utilization of feed, and the feed conversion ratio in Duroc × Landrace × Yorkshire crossbreds [33,34]. Synthetic zeolite in the amount of 0.5% in the diet of fattening pigs (Landrace × Yorkshire × Duroc) did not affect weight gain or carcass quality [35]. In the present study as well, the addition of various sorbents in the two experiments did not significantly influence the carcass value parameters of the pigs. The positive effects on production parameters in animals, therefore, depend on the type of sorbent used (natural or synthetic), [36], its purity, and above all its structure and physicochemical properties, and the level of supplementation in the diet [37]. There are few papers, however, presenting research on the effect of sorbents on the physicochemical properties of pork. 

The average chemical composition of the two skeletal muscles of the pigs from experiments 1 and 2 was similar to the values reported for the Polish population of pigs in commodity production [38,39]. Islam et al. [35] found that the addition of 0.5% zeolite to feed for crossbred pigs (Landrace × Yorkshire × Duroc) had no significant effect on the proximate chemical composition of the *longissimus* muscle (the loin). In comparison with the present study (MLL, Table 4), the authors report lower content of moisture (70.23%) and ash (1.16%), but higher content of protein (26.06%) and fat (2.55%).

By measuring the pH of muscle tissue, it is possible to diagnose potential quality defects in pork. An appropriate pH resulting from post-mortem glycogenolysis ensures favorable sensory and technological properties, including an attractive color, tenderness, palatability, and WHC [40]. Case-ready pork should have a final pH (24 and 48 h post-mortem) ranging from 5.50 to 5.80 [38]. In characterizing the parameters of the technological quality of the meat, it should be stressed that the low initial pH of the muscle tissue of the pigs in experiment 1, in both the control and experimental groups (MLL 5.92–6.01 and MSM 6.03–6.09), was associated with unfavorable atmospheric conditions (high ambient temperature) during transport of the experimental animals to the slaughterhouse (in June) and was not genetically determined. At 24 and 48 post-mortem, the pH of the MLL was about 5.45 and that of the pH was ≥5.5. It should be emphasized, however, that in no case was the final pH of the meat below 5.4, the typical level for acid meat [38]. Nevertheless, based on the values at 24 h post-mortem for pH (<5.5), L* (>50), and DL (>5.0%), [41], the MLL can be considered to show symptoms of the PSE defect, irrespective of the feeding group [42]. Moreover, the greater L* of the MLL may also have been influenced by the degradation of muscle proteins, which depends directly on low final pH, causing increased light dispersion [43]. These unfavorable changes were not observed in the MSM, most likely due to differences in the composition of muscle fibers in the two muscles [44]. The values of the parameters tested in the skeletal muscles of the pigs in all groups in experiment 2 (Table 4) also do not indicate any quality defects in the meat.

Kim et al. [33] reported increasing pH values (5.62, 5.65, and 5.82; *p* < 0.05) for the loin muscle of Landrace × Yorkshire × Duroc fattening pigs with increasing shares of zeolite in their feed (1%, 2% and 4%, respectively), while the pH in the control group was 5.58. In the case of instrumental color parameters, the authors obtained significantly lower L* values (48.81, 47.67, and 47.38) in pigs receiving a higher zeolite supplement (1%, 2%, and 4%, respectively) than in the control group (51.31), i.e., the surface of the meat became darker. This is in contrast to the results of the present study. The values for the other color parameters (redness and yellowness) were not significantly different, although a downward trend was observed—from 9.21 to 7.25 for a* and from 6.12 to 5.33 for b*. For these parameters, the values in the experimental groups (A and B) were higher than in the control group (C2).

Among the other physicochemical properties, the WHC of the MLL was more favorable in the experimental pigs in experiment 2 (Table 2), whose muscles had lower DL. Despite the differences observed, the TBARS value was relatively low, as aroma defects in pork are detectable within a range from 0.5 to 1.0 mg MDA/kg of meat [45]. The higher oxidative stability (lower TBARS) noted in both muscles of the pigs fed with either the addition of biochar (experiment 1) or sorbents A and B (experiment 2) may have been linked to the lower concentration of haem proteins, containing Fe and exhibiting pro-oxidant activity [46]. Shurson et al. [37] observed a linear decrease in iron retention (*p* < 0.05) in pigs fed with increasing amounts of zeolite A (0%, 1%, 2%, and 3%). The authors explain that zeolite A can partially impair the absorption of amino acids, and these in turn form ligands with iron, which are chelating factors in the transport of Fe through the cells of the mucosa for absorption. In this way, Fe absorption is impaired as well, and thus its retention in the body is reduced. This effect was not observed in the case of administration of clinoptilolite in the amount of 2.5%, 5%, and 7.5%. 

The tenderness of the meat (from both skeletal muscles), expressed as shear force in the W–B test, was significantly varied in experiment 1; for the MLL it ranged from 27.42 to 37.93 N and for the MSM from 37.33 to 57.47 N. In experiment 2, the shear force did not differ significantly between the experimental groups in the skeletal muscles, ranging from 47.62 to 55.13 N for MLL and from 65.50 to 77.64 N for MSM. Iwańska et al. [47], taking into account different tenderization processes, proposed the following classification for pork tenderness in terms of W-B shear force (in N/cm^2^): very tender <30, tender 30–45 N, tough 60–90 N, and very tough >90 N. Adopting this classification, the meat in the present study can be classified as tender for both skeletal muscles in experiment 1, while in experiment 2 the MLL was classified as intermediate and the MSM as tough. It should be noted that this level had been reached by 48 h post-mortem and did not include the aging process.

## 5. Conclusions

It can be concluded from the results of the study that the use of natural sorbents as feed additives for pigs has no negative effects on the physicochemical properties of the muscle tissue or its potential suitability for use as case-ready meat or the production of processed meat products. The results indicate that the sorbents used are a safe ingredient in the diet of pigs, as indicated by meat parameters such as optimum pH and water activity and high oxidative stability (TBARS). At the same time, there is a need to continue this line of research, taking into account the meat aging process, packaging, storage time at various temperatures, the quality of processed meat products, and the relationships linking the future production goals of pig farming and processing potential in the meat industry with current climate policy.

## Figures and Tables

**Table 1:** Ingredients and chemical composition (%) of feed for pigs.

Ingredient	Amount (%)	
	Grower	Finisher
Barley, 12.2% CP	32.5	39.8
Wheat, 12.1% CP	24.0	-
Triticale, 11.4 % CP	10.0	30.0
Soybean meal, min. 46.5% CP	12.3	9.0
Maize, 9.46% CP	10.0	10.0
Rapeseed meal, over 3%	3.5	4.0
Mineral mixture 2.5%	2.5	2.5
Soybean oil	1.7	1.4
Chalk	1.0	1.1
JRS Arbocel RC	0.5	0.3
ULTRACID P4 PLUS Nutriade	0.3	0.2
DSM RONOZYM WX VP	0.1	0.1
MYCOFIX + 3E	0.1	0.1
Sorbent A/B/D	1.5/1.5/0.5	

All cereals used in balancing the feed ration were enriched with an enzyme complex—non-starch polysaccharides (NPS) and phytase. CP—crude protein.

**Table 2:** Nutrient content in 1 kg grower feed 40–70 kg and finisher feed >70 kg body weight (BW); experiments 1 and 2.

Nutrient	Content		Requirement		% Met	
	Grower	Finisher	Grower	Finisher	Grower	Finisher
Dry matter, g	762	765	-	-	-	-
Metabolizable energy, MJ	13.2	13.0	13.2	13.0	100	100
Lysine, g	10.3	9.48	10.1	9.00	103	105
Methionine, g	2.80	2.67	3.02	2.70	93	99
Methionine + cystine, g	5.92	5.74	6.03	5.40	98	106
Tryptophan, g	1.97	1.86	1.91	1.70	103	109
Threonine, g	6.49	6.04	6.53	5.85	99	103
Crude protein, g	168	157	168	155	100	101
Calcium, g	8.10	8.32	8.12	8.00	100	104
Total phosphorus, g	5.81	5.79	4.87	4.50	119	129
Digestible phosphorus, g	1.47	1.45	2.64	2.00	56	72
Sodium, g	1.79	1.78	1.73	1.70	104	105
Fiber, g	43.8	46.8	43.1	43.0	102	109
Magnesium, g	1.68	1.40	64.0	65.0	3	2
Manganese, mg	98.6	98.1	40.0	40.0	246	245
Iodine, mg	2.46	2.44	0.200	0.200	1232	1 220
Copper, mg	23.5	23.3	17.5	17.5	134	133
Iron, mg	172	169	80.0	80.0	215	211
Zinc, mg	101	101	100	100	101	101
Selenium, mg	0.520	0.505	0.100	0.100	520	505
Vitamin A, IU	6 500	6 500	6 500	6 500	100	100
Vitamin D_3_, IU	2 000	2 000	1 250	1 250	160	160
Vitamin E, mg	90.1	91.2	80.0	80.0	113	114
Vitamin K_3_, mg	2.33	2.33	1.25	1.25	186	186
Vitamin B_1_, mg	6.34	6.37	1.00	1.00	634	637
Vitamin B_2_, mg	7.73	7.51	4.00	4.00	193	188
Vitamin B_6_, mg	8.40	8.14	2.25	2.25	373	362
Vitamin B_12_, mcg	0.391	0.031	20.0	20.0	2	-
Biotin, mg	0.232	0.231	-	-	-	-
Folic acid, mg	1.43	1.47	0.75	0.75	190	196
Nicotinic acid, mg	77.4	76.7	25.0	25.0	310	307
Pantothenic acid, mg	24.1	23.6	14.0	14.0	172	168
Choline, mg	1 466	1 355	150	150	977	903
Linoleic acid, mg	2 663	3 260	-	-	-	-
Sugar, g	33.9	33.3	-	-	-	-

**Table 3:** Intrinsic properties of the *musculus Longissimus lumborum* (MLL) and *musculus semimembranosus* (MSM), (mean ± SD).

June	MLL		MSM	
	C1 (*n* = 6)	Sorbent D	C1 (*n* = 6)	Sorbent D
		(Biochar), (*n* = 6)		(Biochar), (*n* = 6)
pH_1_	6.01 ± 0.32	5.92 ± 0.24	6.09 ± 0.40	6.03 ± 0.14
pH_24_	5.45 ± 0.03	5.44 ± 0.06	5.49 ± 0.06	5.50 ± 0.03
pH_48_	5.43 ± 0.03	5.46 ± 0.03	5.52 ± 0.06	5.53 ± 0.02
a_W_	0.948 ^A^ ± 0.001	0.957 ^B^ ± 0.005	0.952 ± 0.005	0.954 ± 0.004
L*	55.89 ^A^ ± 0.76	59.34 ^B^ ± 3.15	48.13 ± 3.07	48.74 ± 2.69
a*	18.93 ± 0.58	18.30 ± 0.90	21.19 ^b^ ± 0.70	20.35 ^a^ ± 1.13
b*	6.33 ± 1.23	7.02 ± 0.90	6.20 ± 1.43	5.40 ± 0.98
Pigments (µg/g)	45.80 ^B^ ± 5.29	37.20 ^A^ ± 3.22	62.90 ^B^ ± 1.88	46.20 ^A^ ± 4.00
TBARS	0.228 ^B^ ± 0.027	0.181 ^A^ ± 0.029	0.221 ^B^ ± 0.052	0.159 ^A^ ± 0.041
(mg MDA/kg)				
W-B SF (N)	27.40 ^A^ ± 9.20	37.90 ^B^ ± 6.30	37.30 ^A^ ± 7.40	57.50 ^B^ ± 14.90
W-B SE (mJ)	93.00 ^a^ ± 31.50	117.30 ^b^ ± 28.40	117.70 ^A^ ± 31.70	220.70 ^B^ ± 85.50
Hardness (N)	40.59 ± 12.87	55.93 ± 25.49	74.50 ± 24.05	68.87 ± 30.72
Springiness	0.48 ± 0.06	0.46 ± 0.03	0.50 ± 0.04	0.50 ± 0.05
Gumminess	14.77 ± 5.51	21.86 ± 11.86	26.47 ± 4.89	25.98 ± 9.43
Chewiness	7.29 ± 3.11	9.81 ± 5.33	13.02 ± 2.04	12.60 ± 3.58
DL (%)	5.64 ± 1.65	5.22 ± 0.82	3.09 ± 1.48	2.47 ± 1.14
CL (%)	20.40 ± 2.93	18.51 ± 1.98	21.04 ± 2.11	19.23 ± 2.84
M/T×100	36.98 ± 1.64	36.45 ± 4.34	40.58 ± 4.51	45.08 ± 5.62
G-H (mg)	59.65 ± 1.97	60.82 ± 5.98	57.57 ± 2.52	54.89 ± 9.11
Moisture (%)	72.55 ± 0.18	72.38 ± 0.33	74.12 ± 0.52	74.49 ± 0.68
Protein (%)	23.12 ± 0.75	22.87 ± 0.44	22.56 ± 1.11	22.08 ± 0.71
Fat (%)	2.38 ± 0.81	2.20 ± 0.20	1.65 ± 0.42	1.58 ± 0.75
Ash (%)	1.23 ± 0.08	1.21 ± 0.09	1.22 ± 0.10	1.22 ± 0.09
M:P	3.14 ± 0.10	3.17 ± 0.06	3.29 ± 0.18	3.38 ± 0.12
Energy (kcal)	113.90 ± 5.53	111.20 ± 2.13	105.10 ± 4.26	102.50 ± 7.55

MLL—*m. Longissimus lumborum*; MSM—*m. semimembranosus*; SD—standard deviation; Groups: C1—control; D—group receiving feed with 0.5% of biochar; ^a,b^—Values in rows marked with different letters differ significantly at *p* ≤ 0.05; ^A,B^—Values in rows marked with different letters differ significantly at *p* ≤ 0.01; M:P—moisture to protein ratio; CIE colour parameters: L*—lightness; a*—red; b*—yellow; TBARS—thiobarbituric acid reactive substance; MDA—malondialdehyde; a_W_—water activity; W-B SF—Warner–Bratzler shear force; W-B SE—Warner–Bratzler shear energy; DL—drip loss; CL—cooking loss; M/T—meat sample/total loss × 100; G-H—free water by the Grau–Hamm method [27].

**Table 4:** Physicochemical properties of the *musculus Longissimus lumborum* (MLL) and *musculus semimembranosus* (MSM), (mean ± SD).

December	MLL			MSM		
	C2	Sorbent A	Sorbent B	K2/C2	Sorbent A	Sorbent B
	(*n* = 6)	(*n* = 6)	(*n* = 6)	(*n* = 6)	(*n* = 6)	(*n* = 6)
pH_1_	6.65 ± 0.05	6.47 ± 0.48	6.48 ± 0.05	6.58 ± 0.06	6.71 ± 0.36	6.47 ± 0.12
pH_24_	5.79 ± 0.01	5.69 ± 0.21	5.54 ± 0.05	6.02 ^c^ ± 0.06	5.70 ^b^ ± 0.26	5.55 ^a^ ± 0.03
pH_48_	5.64 ± 0.04	5.44 ± 0.03	5.46 ± 0.02	5.79 ^B^ ± 0.04	5.48 ^A^ ± 0.04	5.49 ^A^ ± 0.02
a_W_	0.947 ± 0.002	0.953 ± 0.006	0.953 ± 0.005	0.949 ± 0.002	0.957 ± 0.007	0.953 ± 0.003
L*	50.58 ^a^ ± 1.15	54.05 ^b^ ± 2.37	53.79 ^b^ ± 1.36	44.56 ^a^ ± 0.75	50.44 ^c^ ± 3.79	48.63 ^b^ ± 2.53
a*	17.51 ± 0.93	18.17 ± 0.93	18.03 ± 0.83	20.56 ± 0.84	19.75 ± 0.86	20.38 ± 0.98
b*	4.21 ± 1.28	5.60 ± 1.84	5.51 ± 1.28	4.09 ± 1.06	5.98 ± 1.57	5.80 ± 1.28
Pigments (µg/g)	53.70 ^C^ ± 1.76	41.80 ^A^ ± 1.26	46.70 ^B^ ± 3.95	64.30 ^B^ ± 3.42	43.70 ^A^ ± 3.82	44.90 ^A^ ± 0.94
TBARS	0.314 ^b^ ± 0.018	0.238 ^a^ ± 0.019	0.280 ^ab^ ± 0.013	0.444 ^C^ ± 0.036	0.280 ^A^ ± 0.012	0.375 ^B^ ± 0.012
(mg MDA/kg)						
W-B SF (N)	47.60 ± 8.80	55.10 ± 21.40	48.30 ± 9.20	65.50 ± 8.70	71.50 ± 21.10	72.60 ± 20.60
W-B SE (mJ)	164.00 ± 28.50	182.00 ± 81.10	155.70 ± 41.80	289.30 ± 41.10	286.20 ± 103.50	285.90 ± 95.30
Hardness (N)	88.90 ± 14.85	96.40 ± 12.86	75.22 ± 12.24	89.15 ± 12.37	89.05 ± 16.15	90.95 ± 33.60
Springiness	0.59 ± 0.05	0.59 ± 0.03	0.60 ± 0.02	0.58 ± 0.03	0.58 ± 0.04	0.56 ± 0.03
Gumminess	34.17 ± 5.83	36.98 ± 2.70	28.90 ± 5.37	37.12 ± 2.64	34.86 ± 5.72	37.21 ± 8.16
Chewiness	20.09 ± 3.32	21.75 ± 0.99	17.38 ± 3.09	21.37 ± 0.52	19.97 ± 2.39	21.11 ± 8.09
DL (%)	3.37 ^b^ ± 0.58	2.25 ^a^ ± 0.13	2.78 ^ab^ ± 0.12	3.78 ± 0.92	3.67 ± 0.44	3.52 ± 0.11
CL (%)	27.04 ± 1.05	29.26 ± 1.61	27.06 ± 1.73	25.67 ± 1.08	26.84 ± 1.32	26.83 ± 2.20
M/T×100	37.34 ± 1.12	38.15 ± 6.59	36.95 ± 2.06	40.58 ± 4.51	45.08 ± 5.62	36.45 ± 4.34
G-H (mg)	50.45 ± 1.59	56.53 ± 4.27	55.91 ± 1.07	57.57 ± 2.52	54.89 ± 9.11	60.82 ± 5.98
Moisture (%)	75.25 ± 0.13	73.68 ± 0.53	74.42 ± 0.10	75.17 ± 0.20	73.41 ± 0.78	74.25 ± 0.19
Protein (%)	21.48 ± 0.57	21.76 ± 1.19	22.22 ± 0.72	21.17 ± 1.46	22.09 ± 1.43	22.12 ± 0.99
Fat (%)	1.08 ± 0.16	1.47 ± 0.42	1.30 ± 0.22	1.81 ± 0.04	2.24 ± 0.32	2.00 ± 0.58
Ash (%)	1.21 ± 0.04	1.25 ± 0.03	1.24 ± 0.03	1.24 ± 0.04	1.17 ± 0.10	1.24 ± 0.08
M:P	3.50 ± 0.09	3.39 ± 0.17	3.35 ± 0.11	3.56 ± 0.26	3.34 ± 0.29	3.36 ± 0.15
Energy (kcal)	95.60 ± 2.78	100.30 ± 3.42	100.60 ± 4.14	101.00 ± 6.21	108.50 ± 3.11	106.50 ± 8.06

MLL—*m. longissimus lumborum*; MSM—*m. semimembranosus*; SD—standard deviation; Groups: C2—control; A and B—groups receiving feed with 1.5% of mixtures (in different proportions) of bentonite–montmorillonite and zeolite–clinoptilolite defined in the patent application; ^a–c^—Values in rows marked with different letters differ significantly at *p* ≤ 0.05; ^A–C^—Values in rows marked with different letters differ significantly at *p* ≤ 0.01; M:P—moisture to protein ratio; CIE color parameters: L*—lightness; a*—red; b*—yellow; TBARS—thiobarbituric acid reactive substance; MDA—malondialdehyde; a_W_—water activity; W–B SF—Warner–Bratzler shear force; W–B SE—Warner–Bratzler shear energy; DL—drip loss; CL—cooking loss; M/T—meat sample/total loss × 100; G–H—free water by the Grau–Hamm method [27].

## Data Availability

All relevant data are within the paper.
